# Ultrasound Attenuation Improves Some Surface Properties of the Probiotic Strain *Lacticaseibacillus casei* ATCC 393

**DOI:** 10.3390/microorganisms11010142

**Published:** 2023-01-05

**Authors:** Irene Giordano, Gianluigi Mauriello

**Affiliations:** Department of Agricultural Science, University of Naples Federico II, 80055 Naples, Italy

**Keywords:** sonication, modulation, fermentation, metabolism, cultivability, adhesion

## Abstract

Ultrasound attenuation has been recently proposed as a tool to modulate probiotic metabolism. The study aimed to characterize the response of the probiotic *Lacticaseibacillus casei* ATCC 393 to sonication. Two ultrasound treatments were tested (57 W, duty cycle 50%, 6 or 8 min). Attenuation was assessed as a pH decrease in MRS broth after 6 and 24 h of incubation at 37 °C. Cultivability was evaluated by plate count immediately after sonication and by growth index on overnight cultures. Surface changes were determined by auto-aggregation, hydrophobicity, biofilm production tests, and by membrane damages. The 6 min treatment induced a temporary attenuation, while a prolongated exposure to sonic waves caused major attenuation effects (ΔpH 0.97 after 24 h). Both sonication treatments affected probiotic cultivability with a significant (*p* < 0.05) reduction of plate counts and an alteration of the growth index. Although auto-aggregation was negatively affected upon sonication, the hydrophobicity and biofilm production were improved with no significant differences (*p* > 0.05) between the sonicated samples. Moreover, sonicated *L. casei* ATCC 393 resulted in increased membrane permeability. These results suggest that ultrasound technology can be successfully used to modulate the *L. casei* ATCC 393 fermentative metabolism and to improve its surface properties.

## 1. Introduction

Hill et al. [1] proposed the most recent definition of probiotics as “live microorganisms that, when administered in adequate amounts, confer a health benefit on the host”. The beneficial role of these microorganisms depends on their ability to promote host metabolic functions, the host immune response, and to normalise the intestinal microbiota composition. To exert such positive effects, probiotics must survive the harsh environment of the gastrointestinal tract, reach the intestine in sufficient amount, and colonize the intestinal mucosa [2]. Intestine colonization occurs through probiotic adhesion to the epithelial cells. Although adhesion is not directly related to health benefits, it leads to an increase in host–bacteria interactions. In fact, strong adhesive abilities extend the probiotic transit time, preventing a rapid expulsion and resulting in a temporary colonization. The adhesion mechanism also promotes in situ action of microbial metabolites, such as bacteriocins and short-chain fatty acids (SCFAs), and of surface-localized immunomodulatory molecules [3].

Probiotics mainly belong to the Lactic Acid Bacteria (LAB), especially to the genus *Lactobacillus*. The extreme heterogeneity of this genus in terms of phenotypic, genotypic, and ecological features led to a recent deep taxonomic differentiation [4]. *Lacticaseibacillus casei* ATCC 393 is a well-known probiotic strain belonging to the *L. casei* group. This probiotic strain has been widely used in food application, especially in dairy products, such as cheese, yoghurt, and fermented milk [5,6,7]. Several health promoting activities have been highlighted: regulation of intestinal microbiota [8], reducing risk of osteoporosis [9], anticancer activity, pro-apoptotic and anti-proliferative effects [10]. Recently, Abdel-Hamid et al. [11] identified bioactive peptides produced by *L. casei* ATCC 393 in fermented milk.

The concept of healthy food for healthy life leads food companies and scientists towards the formulation of new probiotic foods. Plant and fruit-based beverages are becoming intensively studied as an alternative to dairy products as probiotic carriers [12]. In the development of probiotic foods, great attention is paid to consumers’ demands for products that are not only healthy and safe, but also tasty and pleasant [13]. The metabolization of food components by probiotics and the release of bacterial metabolites into the beverage can result in changes of its chemical, physical, and sensory characteristics, affecting consumer general acceptance. Therefore, the main challenge in the production of new probiotic foods is to control the food–probiotic interactions. The concept of attenuation has been applied to starter cultures to control and/or accelerate the ripening phase in cheese production [14]. More recently, attenuation has been proposed as a technological approach to modulate the metabolism of probiotic microorganisms to avoid acidification and post-acidification and, thus, changes in the sensory profile of the probioticated food [15,16].

Physical, chemical, and mechanical treatments have been applied to modulate microbial performance. The thermal shock treatment, both at high and low temperature, is the classic approach used for this purpose. On the other side, the chemical treatments include the use of enzymes, such as lysozyme, and different types of solvents. Genetic engineering can also be applied to produce mutant strains with desired features. High-pressure homogenization, microfluidization, and sonication are emerging technologies that have been studied as metabolic attenuation strategies [17].

Ultrasound, as other non-thermal technology, has gain much attention in the last decade as a means to control the metabolism of microbial cells. It is an alternative, cheap, and sustainable method, which can be tailored according to the treatment goals. The microorganism response depends on the intensity of chain reactions originating from one main phenomenon, namely cavitation. The collapse of cavitation bubbles induces mechanical, thermal, and chemical damages. Shock waves, liquid microjets, microstreaming and high shear forces are responsible for physical damages of the cell wall and membrane. Hot spots formation contributes to local damage. In addition, this leads to the generation of free radicals that interact with the components of the cell membrane as well as with intracellular molecules causing their oxidation [18,19]. The biological effects of ultrasound on microbial cells can be categorized as stimulation, inactivation, or destruction. However, very little is known about the modulation of probiotic’s activity and of the attenuation effect.

Thus, the present paper aimed to study ultrasound treatment on the probiotic *Lacticasebacillus casei* ATCC 393 in terms of attenuation of the fermentative metabolism, cultivability, and adhesion ability.

## 2. Materials and Methods

### 2.1. Probiotic Culture and Cell-Suspension Preparation

The probiotic strain *L. casei* ATCC 393 was purchased by the American Type Culture Collection and was used to evaluate the potential of sonication as attenuation technology. *L. casei* ATCC 393 was cultured in MRS broth (OXOID Ltd., Basingstoke, Hampshire, UK) for 24 h at 37 °C. For the sonication tests, the resulting culture (30 mL) was centrifuged (ALC^®^ Centrifuge PK130, Thermo Fisher Scientific, Waltham, MA, USA) at 6000× *g* for 10 min, washed once and resuspend in an equal volume of deionized water to reach a concentration of 10^9^ CFU/mL [16].

### 2.2. Experimental Design of Sonication Treatment

**Figure 1:**
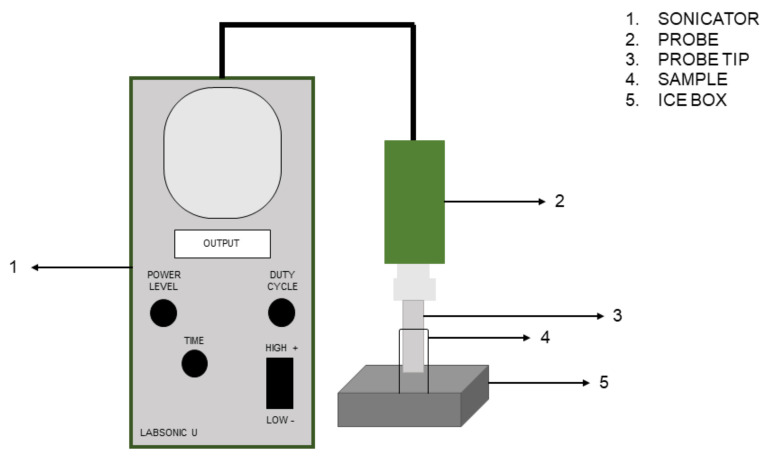
Schematic representation of sonicator LABSONIC U (B. Braun, Melsungen, Germany).

Sonication of cell suspension was carried out by using the LABSONIC U (B. Braun, Melsungen, Germany) working at 20 kHz frequency and equipped with the 50 mL cup probe. Before and after sonication, the probe was cleaned with 70% ethanol. The main parameters of sonication treatment were power level, duty cycle (DC) (the percentage of time during which the ultrasound signal is “on”), and time. Based on our previous work [20], two combinations of the three parameters were chosen: 57 W, 50% DC, 6 min (LC_S6); 57 W, 50% DC, 8 min (LC_S8). The mean efficiency of the probe varied from 77% to 84%. During sonication, the samples were kept in an ice box to avoid an excessive increase in temperature. Figure 1 shows a schematic representation of the equipment used.

### 2.3. Acidifying Capabilities

Attenuation of fermentative metabolism was assessed by inoculating (1%) the sonicated cell suspension and the untreated probiotic in MRS broth. Samples were incubated at 37 °C and the pH was monitored using a digital pH-meter (BENCH METER-pH 80) after 6 and 24 h of incubation. Collected data were reported as pH decrease (ΔpH) [16].

### 2.4. Probiotic Cultivability

Immediately after the ultrasound treatment, 1 mL of sonicated and not-sonicated cell suspensions were serially diluted in quarter-strength Ringer solution and plate-counted on MRS Agar (37 °C, 48–72 h). Furthermore, sonicated and not-sonicated cells were inoculated in MRS broth and incubated at 37 °C for 24 h. The population level at this time was measured by measuring the optical density (OD) at 600 nm of the culture in absorbance (A) modality (Eppendorf BioSpectometer^®^ basic). Moreover, a standard growth curve of the control culture was preliminarily determined by measuring OD and viable counting after 0, 2.5, 4, 6, 12, 16, 18, 20 and 24 h. The collected data were reported as growth index (*GI*) as shown in Equation (1):

(1) $$GI=\frac{A_{US}}{A_{C}}\times 100$$

*A_US_* is the absorbance of sonicated samples and *A_C_* is the absorbance of the control (untreated bacteria).

As suggested by Bevilacqua et al. [21]:

− *GI* < 25% means complete inhibition;
− 25% < *GI* < 75% means partial inhibition;
− *GI* > 75% means no inhibition.

Therefore, probiotic cultivability was evaluated based on these two parameters.

### 2.5. Characterization of Cell Surface Properties

*L. casei* ATCC 393 surface properties were characterized based on probiotic auto-aggregation capabilities, cells hydrophobicity, and biofilm formation.

#### 2.5.1. Auto-Aggregation

An auto-aggregation test was carried out as per Fonseca et al. [22] with slight modifications. Sonicated and not-sonicated cell suspensions were diluted to an OD_600_ 0.600 ± 0.200. The absorbance was read at t_0_ (*A*_0_), the samples were vortexed for 10 s and then incubated at 37 °C for 5 h. After incubation, an aliquot was taken without shaking the sample and the absorbance was read again (*A_t_*). Data were reported as auto-aggregation percentage (*AA*%) through the Equation (2):

(2) $$AA\%=\left[\frac{\left(A_{0}-A_{t}\right)}{A_{0}}\right]\times 100$$

#### 2.5.2. Hydrophobicity

Probiotic hydrophobicity was defined as a cell’s affinity to iso-octane and evaluated through the Microbial Adhesion to Hydrocarbon Test (MATH). The protocol was developed as described by Fonseca et al. [22] with some modification. The samples were diluted as described above and after reading the absorbance at t_0_ (*A*_0_), 1 mL of iso-octane was added to 3 mL of cell suspensions. The aqueous and apolar phase were mixed by vortex for 2 min and then incubated at 37 °C for 1 h. The hydrophobicity percentage (*HY*%) was calculated as described in Equation (3):

(3) $$HY\%=\left[\frac{\left(A_{0}-A_{t}\right)}{A_{0}}\right]\times 100$$

where *A_t_* is the absorbance of the aqueous phase after 1 h of incubation.

#### 2.5.3. Biofilm Formation Assay

Biofilm formation was evaluated using a colorimetric assay as previously described by Chen et al. [23] and Stepanović et al. [24] with major modifications. Sonicated cell suspensions were diluted to an OD_600_ 1.000 ± 0.200 and 1.5 mL was added into each well of a sterile 24-well flat-bottom microtiter plate. After 24 h of incubation at 37 °C, the wells were washed three times with phosphate saline buffer (PBS). The adherent cells were fix by storing the plate at 60 °C for 60 min and then stained with 1.5 mL of 10% crystal violet solution. After 15 min, the excess stain was removed by washing the plate with deionized water. The plate was air dried and then an equal volume of glacial acetic acid solution (33%) was added to each well to dissolve the stain. The experiment was performed in triplicate and the absorbance of wells containing sterile MRS broth was used as a negative control. The absorbance was read at 570 nm. Data interpretation was conducted by establishing a low cut-off (ODc) as 3 × standard deviation above the mean values of the control wells. The strains were classified into the following categories: no biofilm producer (OD ≤ ODc); weak biofilm producer (ODc < OD ≤ 2ODc); moderate biofilm producer (2ODc < OD ≤ 4ODc); and strong biofilm producer (OD > 4ODc).

### 2.6. Membrane Permeability

Membrane damage induced by ultrasound treatments was evaluated quantifying the release of intracellular components [25]. Briefly, cell suspensions for both sonicated and not-sonicated samples were centrifuged at 8000× *g* for 15 min. The supernatant was collected, and the absorbance was read at 260 nm (proteins) and at 280 nm (nucleic acids) using an UV-Vis spectrometer (Thermo Fisher Scientific, Waltham, MA, USA). Results were reported as absorbance increase (*AI*%) and elaborated by Equation (4):

(4) $$AI\%=\left[\frac{\left(A_{US}-A_{C}\right)}{A_{C}}\right]\times 100$$

where *A_US_* is the absorbance of sonicated samples and *A_C_* is the absorbance of not-sonicated sample used as control.

### 2.7. Statistical Analysis

All the analyses were repeated three times (*n* = 3) and results were reported as the mean of the experiments performed with the standard deviation. Data were analysed through two-way ANOVA and Student’s *t*-test (SPSS) to ascertain significant differences between the means. Significance was declared at *p* < 0.05.

## 3. Results and Discussion

### 3.1. Fermentative Metabolism Attenuation

Table 1 shows the results of ultrasound-induced attenuation.

After 6 h of incubation there were significant differences (*p* < 0.05) between the control and sample LC_S6. However, the combination 57 W/50% DC/6 min temporary attenuated *L. casei* ATCC 393 fermentative metabolism. In fact, after 24 h of incubation no significant differences (*p* > 0.05) were found for LC_S0 and LC_S6. Moreover, after 6 h of incubation the two sonication treatments had a similar modulation effect (*p* > 0.05), and instead significant differences (*p* < 0.05) were found after 24 h. The treatment at 57 W/50% DC/8 min resulted in a complete attenuation of the probiotic fermentative metabolism. After 24 h of incubation, the pH decrease was 2.16 ± 0.18 for LC_S0 and 0.97 ± 0.14 for LC_S8 (*p* < 0.05). In the development of new probiotic beverages there are two main probiotication strategies: fermentation or addition. Attenuated probiotics can be an alternative to formulate functional drinks and avoid physical–chemical and sensory changes induced by the beneficial bacteria. Results of attenuation experiments imply that by increasing the sonication intensity there is a stronger attenuation effect. The LC_S6 was found to restore its metabolism after 24 h of incubation. Interestingly, in the same period, minimal acidification occurs when the probiotic is exposed to the treatment for 8 min. Similar findings have been observed by Racioppo et al. [16]. After 6 h of incubation, they found an increase in the attenuation effect by increasing the power and the duration of the ultrasound treatment. However, after 24 h of incubation no significant difference was found between the control and the sonicated samples. Surprisingly, unlike previous studies [16,18,26], we found a good and long-lasting response of *L. casei* ATCC 393 to ultrasound attenuation. We hypothesize an alteration of the correct functionality of the microbial enzymes involved in the sugar transport systems and sugar consumption. Free radicals formed by implosion of cavitation bubbles interact with bacterial extracellular and intracellular molecules, leading to their oxidation. OH· attacks electron-rich sites, such as the double bond of the amino acids, thus inhibiting the specific function of the corresponding enzyme [19]. The results obtained by Liao et al. [27] are in support of this hypothesis. They found intracellular chemical damage as ATP levels decrease and DNA ruptures, and suggested that the radical oxygen species (ROS) can be injected into the cells with cavitation microjets.

Although the collected data highlight the central role of treatment exposure duration in cell response, further observations are needed to understand the exact mechanism by which attenuation occurs.

### 3.2. Microbial Growth: Plate Count and Growth Index

Results of viable count and GI after exposure to the sonication are shown in Table 2.

The 6 and 8 min treatments caused approximately 1 and 3 Log reduction (*p* < 0.05), respectively. The growth index analysis shows that there was no growth inhibition for sample LC_S6 (GI > 75%). In contrast, complete growth inhibition (GI < 25%) was recorded in the case of LC_S8. By comparing A_US_ to the standard growth curve, the population level of LC_S6 and LC_S8 after 24 h of growth in MRS was estimated at 9.13 and 7.82 Log CFU/mL, respectively. Therefore, probiotic cultivability is affected when subjected to sonication. Ultrasound treatments were aimed to attenuate the probiotic fermentative metabolism without negatively affecting its viability. The results obtained showed that the ability of the three samples to form colonies in plates was markedly different. However, this parameter is not sufficient to evaluate the cell viability. In fact, although the growth and division are widely accepted as synonymous with viability, other parameters contribute to the definition of a viable cell, such as the presence of an intact membrane and metabolic activity. It is known that bacteria can enter in a viable but non-culturable (VBNC) state if exposed to stressful treatments or environments [28]. Therefore, we can suppose that ultrasound treatments applied on *L. casei* ATCC 393 induce the VBNC state. However, further analysis regarding cytoplasmic membrane function, metabolic activity, and genetic functionality is required for a proper definition of VBNC status. Moreover, it has been proven that microbial cells can restore normal functions or can resuscitate, under suitable conditions [29]. Results obtained from the growth index analysis suggest that LC_S6 resuscitates. Perhaps, the more intense treatment may have caused LC_S8 inactivation. However, the inactivated probiotics, properly called postbiotics, are able to exert similar or improved beneficial effects on the host than those exerted by the corresponding active cells. Recently, Brandão et al. [30] found that in rats fed with a high fat diet, the consumption of ultrasound inactivated *Lacticaseibacillus casei* 01 can improve hypertension and cholesterol levels. Therefore, reduction or inhibition of growth is not directly related to the health properties of ultrasound-treated cells.

### 3.3. Cell Surface Properties

Cell surface properties are summarized in Table 3.

*L. casei* ATCC 393 exhibited approximately 20-fold higher auto-aggregation than LC_S6 and LC_S8 samples. In addition, by increasing the duration of the treatment there was a significant (*p* < 0.05) reduction of the auto-aggregation capability. The percentage of hydrophobicity was 6.29, 11.68 and 15.01 for LC_S0, LC_S6 and LC_S8, respectively. Statistical analysis revealed significant differences (*p* < 0.05) between the control and the sonicated samples; however, no significant differences (*p* > 0.05) were found between the two sonicated samples. In addition, LC_S6 and LC_S8 showed a stronger biofilm production compared to the parental strain *L. casei* ATCC 393. Auto-aggregation, MATH and biofilm production are preliminary tests commonly applied for an initial assessment of the adhesive capacities of microbial strains. Adhesion mechanism involves specific and non-specific interactions between two surfaces that depend on the composition of both surfaces [3]. Few studies in literature have analysed the impact of ultrasound on bacterial surface properties. For the tested strain, a negative correlation between sonication treatment and auto-aggregation was found. Different findings have been previously reported [31]. As a matter of fact, when three probiotic *Lactobacillus* strains were exposed to the ultrasound treatment for more than 20 min, they were able to form aggregates with a *Streptobacillus*-like morphology. The authors suggested that ultrasound can stimulate and activate surface proteins involved in the auto-aggregation mechanism. Although our results are not in agreement with previous findings, it is not possible to directly compare the results of studies conducted in different ways on different bacterial strains.

Upon sonication, the probiotic hydrophobicity was increased, and biofilm production was improved. Adhesion to the hosts’ intestinal epithelium surface molecules (IESM) is an important feature for the survival and the persistence of beneficial microorganisms along the human gastrointestinal tract [32]. High hydrophobicity enables better binding to epithelial cells, improving the contact between probiotics and the mucus layer [33]. Racioppo et al. [16] reported similar results on hydrophobicity for the strain *Limosilactobacillus reuteri* DSM 20016. Instead, on the *Lactiplantibacillus plantarum* L12 ultrasound attenuation exerted the opposite effect, and no impact on the hydrophobicity of *Bifidobacterium longum* Bb46 and *Bifidobacterium infantis* Bb02 was found. Increased hydrophobicity for ultrasound-treated *Propionibacterium freudenreichii* subsp. *freudenreichii* DSM 20271 and *Acidipropionibacterium jensenii* DSM 20535 was also reported by Bevliacqua et al. [25]. Moreover, they found a positive correlation between an increased hydrophobicity and an increased biofilm production. This highlights that biofilm formation is affected by hydrophobicity.

Our results suggest that ultrasound alters the surface structure of *L. casei* ATCC 393 and thus affects its normal function and physiological activities. This might explain the distinct adhesive properties of the sonicated strains. In addition, comparing our data with those in the literature, it is evident that the response to ultrasound treatment is strain and species specific.

### 3.4. Structural Alteration of Cell Membrane

The effect of ultrasound on the cell membrane structural characteristics was also studied. Table 4 reported the results of the alteration of the membrane permeability upon sonication.

LC_S6 showed an increase of 216 and 140% at 260 and 280 nm, respectively (*p* < 0.05). Instead, for LC_S8 the increase was 256% at 260 nm and 165% at 280 nm (*p* < 0.05). The cell membrane is the first site of the cell affected by ultrasound. Membrane structure is impaired due to the formation of pores and ruptures, and it is also weakened due to thinning phenomena [19]. Membrane damage was indirectly measured by quantification of intracellular components released upon ultrasound treatment. Sonication increased the absorbance at both 260 and 280 nm; therefore, sonicated *L. casei* ATCC 393 resulted in an increased membrane permeability and high cellular mechanical damage. Our results are consistent with those reported previously [25]. Interestingly, they found that 24 h after the ultrasound treatment the strains partially restored the membrane integrity.

The cell membrane exerts multiple functions, both structural and functional, regulating nutrient transport and metabolites release, communication between the intracellular and extracellular environment, and cell-to-cell communication. Therefore, the cell membrane plays a key role in cell viability and function. Our results showed that ultrasound treatments affect its structure and, therefore, its role. Therefore, high control of process parameters is necessary to achieve the desired level of cell permeability and avoid cell death.

## 4. Conclusions

In this study, sonication was applied on probiotic *L. casei* ATCC 393 as an attenuation strategy. Our results showed that the attenuation effect depends on the intensity of the ultrasonic treatment. While the increase in intensity led to a prolonged attenuation effect, it also caused a significant change in the growth and cultivability of the probiotic strain. Ultrasound treatment also altered adhesion properties of the probiotic by negatively affecting the auto-aggregation abilities and positively affecting the hydrophobicity and biofilm production. In addition, cavitation led to the formation of pores and fractures in the membrane, thus altering its permeability.

The control of microbial performances, especially related to sugar metabolism, is of great interest in the field of probiotic foods. Attenuation could be a promising way for the formulation of probiotic food and beverages with stable sensory characteristics and long shelf-life. In addition, ultrasound-induced attenuation could provide, on the one hand, more stable probiotic products and, on the other hand, higher healthy potential due to the enhanced cell adhesion ability.

Although the collected data are interesting, it is important to underline some limitations of this study. The obtained results cannot be generalized and depend on the experimental design, the device, and the probiotic strain. Moreover, the effect of sonication on microbial cells depends not only on mechanical damage but also on chemical damage. Therefore, it is necessary to study the contribution of chemical damage for a detailed understanding of the phenomenon.

## Figures and Tables

**Table 1:** Decrease in pH of MRS broth inoculated with cells of sonicated and not-sonicated *Lacticaseibacillus casei* ATCC 393, after 6 and 24 h of incubation at 37 °C. LC_S0: not-sonicated cells, control; LC_S6: treatment at 57 W with 50% DC for 6 min; LC_S8: treatment at 57 W with 50% DC for 8 min.

Sample	Time of Incubation (h)	
	6	24
LC_S0	0.38 ± 0.05 ^a^	2.16 ± 0.18 ^a^
LC_S6	0.06 ± 0.02 ^b^	1.91 ± 0.12 ^a^
LC_S8	0.02 ± 0.02 ^b^	0.97 ± 0.14 ^b^

Data are reported as mean values ± standard deviation (*n* = 3). Different letters in the same column indicate that the differences between the samples are significant (*p* < 0.05).

**Table 2:** Plate counts (Log CFU/mL) and GI of sonicated and not-sonicated cells of *Lacticaseibacillus casei* ATCC 393. LC_S0: not-sonicated cells, control; LC_S6: treatment at 57 W with 50% DC for 6 min; LC_S8: treatment at 57 W with 50% DC for 8 min.

Sample	Log CFU/mL	GI
LC_S0	9.33 ± 0.06 ^a^	
LC_S6	8.61 ± 0.16 ^b^	>75%
LC_S8	6.47 ± 0.07 ^c^	<25%

Results are reported as mean values ± standard deviation (*n* = 3). Different letters indicate that the differences between the samples are significant (*p* < 0.05).

**Table 3:** Surface properties of sonicated and not-sonicated cells of *Lacticaseibacillus casei* ATCC 393. LC_S0: not-sonicated cells, control; LC_S6: treatment at 57 W with 50% DC for 6 min; LC_S8: treatment at 57 W with 50% DC for 8 min.

Sample	Auto-Aggregation (%)	Hydrophobicity (%)	Biofilm Production
LC_S0	23.98 ± 1.32 ^a^	6.29 ± 0.68 ^a^	Weak
LC_S6	3.39 ± 0.78 ^b^	11.68 ± 2.65 ^b^	Strong
LC_S8	0.65 ± 0.26 ^c^	15.01 ± 1.59 ^b^	Strong

Data are reported as mean values ± standard deviation (*n* = 3). The results of the biofilm production assay derived from three different replicates. Different letters in the same column indicate that the differences between the samples are significant (*p* < 0.05).

**Table 4:** Absorbance increase (%) at 260 and 280 nm of the supernatant after the sonication treatment of *Lacticaseibacillus casei* ATCC 393 cells. LC_S6: treatment at 57 W with 50% DC for 6 min; LC_S8: treatment at 57 W with 50% DC for 8 min.

Sample	Wavelength (nm)	
	260	280
LC_S6	216 ± 8.16 ^a^	140 ± 10.00 ^a^
LC_S8	256 ± 10.69 ^b^	165 ± 9.64 ^b^

Data are reported as mean values ± standard deviation (*n* = 3). Different letters in the same column indicate that the differences between the samples are significant (*p* < 0.05).

## Data Availability

Not applicable.
